# The complete mitochondrial genome of the *Rana kukunoris* (Anura: Ranidae) from Inner Mongolia, China

**DOI:** 10.1080/23802359.2019.1710591

**Published:** 2020-01-14

**Authors:** Jifei Wang, Zongzhi Li, Hui Gao, Zhensheng Liu, Liwei Teng

**Affiliations:** a Helan Mountains National Nature Reserve of Ningxia, Yinchuan, China;; b College of Wildlife and Protected Area, Northeast Forestry University, Harbin, China;; c Key Laboratory of Conservation Biology, National Forestry and Grassland Administration, Harbin, China

**Keywords:** Complete mitochondrial genome, *Rana kukunoris*, amphibian

## Abstract

We determined the whole mtNDA genome of the Plateau brown frog (*Rana kukunoris*) in Helan Mountains, Inner Mongolia. The complete mitochondrial genome consists of 13 protein-coding, 22 tRNA, 2 rRNA genes, and 1 control region (CR), and its total length is 16,644 bp. Three overlaps among the 13 protein-coding genes were found: ATP8/ATP6, ND4L/ND4, and ND5/ND6. The CR is 837 bp in length. The nucleotide composition is 27.49% A, 29.06% T, 14.77% G, 28.68% C. The result of phylogenetic analysis showed that there was close genetic relationship between *R. kukunoris* and *Rana chensinensis*.

The Plateau brown frog (*Rana kukunoris*) is one species of frog in the family Ranidae, endemic to the eastern Tibetan plateau (29–41°N, 93–104°E), it occurs at elevations ranging from 1500 to 4400 m (Fei et al. 2009). In earlier taxonomic revisions, the Plateau brown frog was considered a subspecies of *Rana temporaria* and later a subspecies of *Rana chensinensis*, but now it is considered as a valid species, which was based on morphology and mitochondrial DNA sequences (Xie et al. 2000; Jiang et al. 2002; Fei et al. 2010; McDonough 2014).

The complete mitochondrial genome of the Plateau brown frog was sequenced using muscle tissue gathered at Helan Mountains, Inner Mongolia, China (105°54′55.96″E, 38°52′05.46″N), and stored in College of Wildlife and Protected Area, Northeast Forestry University (No. LW160728). The sequence was submitted to the GenBank with the accession number MN733918.

The total length of the genome is 16,644 bp with a base composition of 27.49% A, 29.06% T, 14.77% G, and 28.68% C. The complete mitochondrial genome consists of 13 protein-coding genes, 2 rRNA genes (12S rRNA, and 16S rRNA), 22 tRNA genes, and 1 control region (CR). The total length of 13 protein-coding genes is 11,328 bp long, all of which are encoded on the same strand except for ND6 in the light strand. Except for ND1 (ATT start codon), COX1 (ATA start codon), and ND4L (GTG start codon), the remaining 10 protein-coding genes initiate with ATG (*ND2, ND3, ND4, ND5, ND6, COX2, COX3, ATP6, ATP8, Cytb*). The total length of all tRNA genes is 1552 bp long and ranging from 66 bp (tRNA-Cys) to 74 bp (tRNA Leu (UAA)). Lengths of two rRNA genes and control region are 929 bp (12 s rRNA), 1578 bp (16S rRNA) and 837 bp (control regions), respectively.

The phylogenetic relationship was inferred by the maximum likelihood method based on the Tamura-Nei model (Tamura and Nei 1993) and conducted in MEGA7 (Kumar et al. 2016) (Figure 1). The bootstrap consensus tree inferred from 1000 replicates is taken to represent the evolutionary history of the taxa analyzed (Felsenstein 1985). The phylogenetic tree appeared that the phylogenetic relationship of Plateau brown frog is very close to *R. chensinensis*.

**Figure 1.**
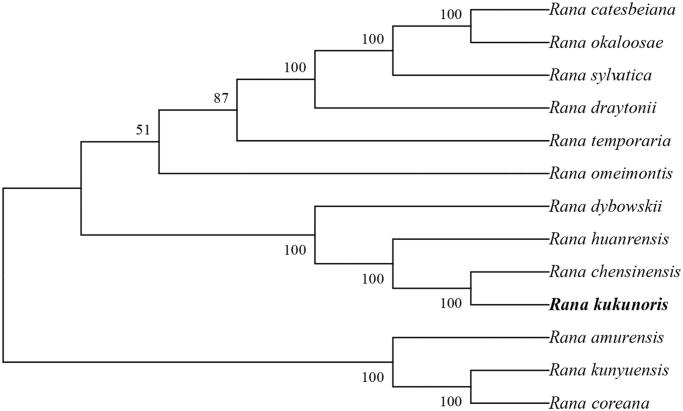
Phylogenetic tree generated using the maximum likelihood method based on complete mitochondrial genomes of 13 species in Anura: Ranidae. GenBank accession numbers: *Rana catesbeiana* (KX686108.1), *Rana okaloosae* (KP013096.1), *Rana sylvatica* (KP222281.1), *Rana draytonii* (KP013110.1), *Rana temporaria* (NC042226.1), *Rana omeimontis* (KU246050.1), *Rana dybowskii* (KF898355.1), *Rana huanrensis* (KT588071.1), *Rana chensinensis* (KF898356.1), *Rana kukunoris* (This study), *Rana amurensis* (MF370348.1), *Rana kunyuensis* (KF840516.1) and *Rana coreana* (KM590550.1).
